# Degradation Behaviors and Mechanism of Nitrile Butadiene Rubber Caused by Insulating Medium C_5_F_10_O

**DOI:** 10.3390/polym15102282

**Published:** 2023-05-12

**Authors:** Congdong She, Fuping Zeng, Liangjun Dai, Long Li, Qiang Yao, Ju Tang

**Affiliations:** 1 School of Electrical Engineering and Automation, Wuhan University, Wuhan 430072, China; 2 State Grid Chongqing Electric Power Research Institute, Chongqing 400022, China; 3 State Key Laboratory of Power Transmission Equipment and System Security and New Technology, Chongqing University, Chongqing 400044, China

**Keywords:** C_5_F_10_O, nitrile butadiene rubber (NBR), compatibility, deterioration, sealing performance, molecular dynamics

## Abstract

C_5_F_10_O is a promising insulating medium in the manufacturing of environmentally friendly gas-insulated switchgears (GISs). The fact that it is not known whether it is compatible with sealing materials used in GISs limits its application. In this paper, the deterioration behaviors and mechanism of nitrile butadiene rubber (NBR) after prolonged exposure to C_5_F_10_O are studied. The influence of C_5_F_10_O/N_2_ mixture on the deterioration process of NBR is analyzed through a thermal accelerated ageing experiment. The interaction mechanism between C_5_F_10_O and NBR is considered based on microscopic detection and density functional theory. Subsequently, the effect of this interaction on the elasticity of NBR is calculated through molecular dynamics simulations. According to the results, the polymer chain of NBR can slowly react with C_5_F_10_O, leading to deterioration of its surface elasticity and loss of inside additives, mainly ZnO and CaCO_3_. This consequently reduces the compression modulus of NBR. The interaction is related to CF_3_ radicals formed by the primary decomposition of C_5_F_10_O. The molecular structure of NBR will be changed in the molecular dynamics simulations due to the addition reaction with CF_3_ on NBR’s backbone or branched chains, resulting in changes in Lame constants and a decrease in elastic parameters.

## 1. Introduction

SF_6_ is a widely used insulating medium in gas-insulated switchgears (GISs) because of its great insulation strength [1]. However, since it is a strong greenhouse gas with a global warming potential (GWP) of 23,500, the application of SF_6_ has been restricted in past decades [2,3]. Gradually reducing or even entirely eliminating the consumption of SF_6_ in the power industry has become a consensus approach in lots of countries and regions [4]. Thus, searching for environmentally friendly substitutes for SF_6_ has been an urgent issue for the future power industry.

In recent years, perfluoro (3-methyl-2-butanone) C_5_F_10_O has been found to be a promising substitute for SF_6_ in medium or low voltage switchgears [5]. The GWP of C_5_F_10_O is only 1, while its insulation strength is 1.93 times that of SF_6_. Series studies on its physical and chemical properties [6], insulation performance [7,8] and electric and thermal decomposition characteristics properties [9,10,11] have been carried out. The results basically prove the feasibility of using C_5_F_10_O mixed with N_2_, CO_2_ or dry air as an insulating medium in environmentally friendly switchgears.

However, compared with SF_6_, which possesses a simple and symmetrical molecular structure, C_5_F_10_O has poorer chemical stability. It is more likely to interact with various materials inside the switchgear under the action of electricity or heat [12]. The interaction can lead to deterioration of various materials in the switchgear, including metal electrodes, solid insulation materials and sealing rubber. As an example, in the study on the compatibility between C_5_F_10_O and copper, after only eight hours of coexistence at 120 °C, micro corrosion marks could be observed on the surface of Cu electrodes, which need to be in contact with C_5_F_10_O throughout the years in a GIS [13]. Meanwhile, long-term exposure to C_5_F_10_O/N_2_ mixture has been reported to corrode the surface and reduce the tensile strength of ethylene propylene diene monomer (EPDM) [14]. The degradation of material properties can significantly impair the performance of GIS, leading to reduced safety and reliability over prolonged operation [15]. Therefore, clarifying the deterioration behaviors of different materials with C_5_F_10_O is the prerequisite for its large-scale engineering application.

In gas-insulated switchgears, rubber rings are utilized in the sealing groove to maintain airtightness within the insulating chamber. The sealing rubber will be in direct contact with C_5_F_10_O throughout the equipment’s lifespan, which can extend up to 30 years. [16]. If the used rubber material is not compatible with C_5_F_10_O, its mechanical properties could degrade over time from prolonged exposure. This degradation could lead to air chamber leakage and weakened insulation strength, ultimately leading to serious failures. However, current research on the degradation of sealing materials in C_5_F_10_O only identifies certain rubber types that may be incompatible with C_5_F_10_O [17]. The process of rubber degradation and the underlying mechanism of its interaction with C_5_F_10_O remain inadequately understood.

To test the compatibility of sealing material and insulating gas, sealed glass tube, headspace bottle and high-pressure sealing tube methods were used in previous studies. [18,19]. Considering the time cost of compatibility experiments, thermal accelerated aging is usually applied to shorten the test time [20]. These methods have been adopted to analyze the failure mechanism of various sealing rubbers in O_2_, SF_6_, C_4_F_7_N and other gas atmospheres [21,22]. Currently there is no recognized standard for the compatibility test procedure; thus, the selection of test conditions needs to be formulated according to the actual working state of the sealing medium.

This paper investigates the degradation behavior of nitrile butadiene rubber (NBR) in C_5_F_10_O, which is commonly used as a sealing material in gas-insulated switchgears. To figure out the effect of prolonged exposure to C_5_F_10_O on the mechanical properties of NBR, a thermal accelerated aging experiment is conducted. The interaction mechanism between NBR and C_5_F_10_O is analyzed with morphology detection, energy spectrum analysis and density functional theory (DFT) calculations. Molecular dynamics (MD) simulations are also employed to explore changes in NBR’s mechanical properties following reactions with C_5_F_10_O. The findings can provide a basis for the selection and maintenance of sealing materials applied to C_5_F_10_O-insulated electrical equipment.

## 2. Experiment Method

### 2.1. Thermal Accelerated Ageing Method

Referring to test procedures of rubber materials specified in ISO 188-2011 [23], a thermal accelerated ageing method for NBR in C_5_F_10_O mixed gas is designed. In previous studies, CO_2_, N_2_ and air have been usually used as the background gas of C_5_F_10_O. While according to the compatibility of common gases with non-metallic materials [24], N_2_ has excellent compatibility with NBR and it does not react with NBR nor cause swelling or weight loss. Thus, the mixture of C_5_F_10_O and N_2_ is chosen to avoid interference of the background gas with the experimental results.

In terms of the ageing temperature, the highest temperature rise of GIS shell is limited to 65 °C (with ambient temperature as the reference value), so the ageing temperature in this experiment is selected as 100 °C. This temperature is much lower than the decomposition temperature of NBR, so it does not change the interaction mechanism, but only exerts the effect of acceleration. In addition, 100 °C is also a suggested ageing condition for rubbers in ISO 188-2011. As a reference, in the thermal accelerated ageing test carried out by Woo C et al., the ageing rate of NBR in the air-oven method at 100 °C is equivalent to about 53 times that at 25 °C [25].

**Figure 1:**
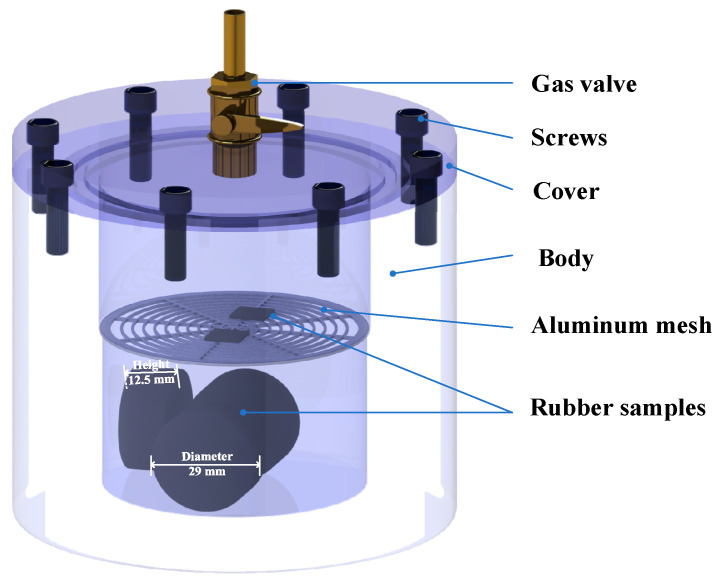
Experimental device and rubber samples used for thermal ageing.

In the experiment, NBR was placed in an airtight container made of aluminum alloy, which was filled with 0.2 MPa 5% C_5_F_10_O/95% N_2_ mixed gas, as shown in Figure 1. A filling pressure of 0.2 MPa is selected to simulate the operating conditions (0.12~0.16 MPa) of real GIS gas chambers. A mixing ratio of 5% is a commonly chosen ratio in previous studies as it is capable of satisfying the required level of electrical strength [5,6]. In the control group, the container is filled with pure N_2_. The container was later put into a constant temperature oven for thermal ageing. The ageing process lasted for 28 days, with 7 days as the experimental interval. The eight groups of samples were independently put into different containers in the experiment. Then, the changes in the mechanical properties, surface morphologies and element content of the NBR before and after the thermal ageing were tested and compared at every experiment interval. In each container, three samples were placed and tested to ensure the repeatability of the results.

### 2.2. Preparation for the NBR Samples

NBR is one of the most commonly used sealing materials for GISs. The sealing rings apply stress on the sealing interface when being compressed in the sealing gap, so that the rubber and the sealing interface can be closely fitted to maintain the airtightness of the equipment. Once the elasticity of the rubber is weakened, the stress on the sealing interface may not be sufficient to withstand the pressure difference in and outside the equipment, which may eventually result in the leakage of the GIS. Hence, the compression modulus is chosen as the indicator of NBR’s sealing performance in this study. The compatibility of NBR and C_5_F_10_O is judged through the compressive modulus before and after the long-term coexistence in the thermal ageing procedure.

The test sample for the compressive modulus is manufactured according to ISO 7743-2017: a cylinder with a height of 12.5 mm and a diameter of 29 mm, as shown in Figure 1 [26]. In addition, sheet-like rubber samples with a thickness of 0.8 mm and a side length of 10 mm were also produced for the morphology and energy spectrum analysis after the compatibility experiment.

**Figure 2:**
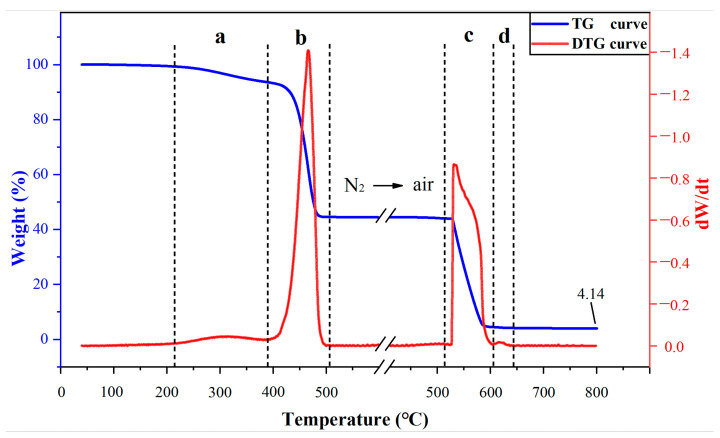
Differential thermogravimetric and thermogravimetric curves of NBR (**a**) ester, (**b**) polymer, (**c**) carbon black, (**d**) CaCO_3_.

The NBR samples tested in this paper are all provided by State Grid Pinggao Group Co., Ltd., Pingdingsha, China. Thermogravimetric analysis on Mettler-Toledo TGA2/DSC3 is used to determine the specific components contained in NBR. During the thermogravimetric analysis, the NBR samples are first heated from room temperature to 600 °C at a rate of 10 °C/min in N_2_ atmosphere, then cooled to 400 °C at the same rate and atmosphere, and finally heated from 400 to 800 °C in air atmosphere [27]. The thermogravimetric (TG) curve and differential thermogravimetric (DTG) curve of NBR are shown in Figure 2.

The weight loss of NBR is divided into four different stages according to the decomposition temperature [27]. The main components and contents of the NBR used in this experiment are listed in Table 1. The most important components of rubber are the carbon black that constitutes the rubber skeleton and the copolymer of butadiene and acrylonitrile, accounting for 88.8% of the total weight. Therefore, when exploring the compatibility mechanism between NBR and C_5_F_10_O, key attention should be paid to the interaction between C_5_F_10_O and these two main components.

## 3. Deterioration Behaviors of NBR Aged in C_5_F_10_O/N_2_ Mixture

NBR has been widely applied as a sealing medium for SF_6_-insulated equipment for many years. A good compatibility between NBR and SF_6_ has already been proved via relevant standards and research. Therefore, in this study, we mainly explain the compatibility of NBR and C_5_F_10_O by comparing the deterioration behavior of NBR in C_5_F_10_O/N_2_ and pure N_2_ atmospheres, without comparing the results to that in the SF_6_ atmosphere.

### 3.1. Compressive Modulus

The compressive modulus of the cylindrical samples was tested every 7 days to study the impact of C_5_F_10_O on the mechanical properties of NBR. The compression stress–strain test was carried out on an INSTRON 7000 universal testing machine made by Instron, Norwood, America. According to [26], the samples were compressed at a rate of 10 mm/min until the strain reached 25%, then released at the same rate, and this process was repeated four times. The stress change during the last compression process is recorded and the compressive modulus was then calculated by the following formula:

(1) $$E_{s}=\frac{F}{A\varepsilon }$$

where *E_s_* is the compressive modulus (MPa), *F* is the compressive stress (N), *A* contributes the cross-sectional area of the sample (mm^2^). *ε* is the compressive strain, when calculating the compressive modulus, *ε* is usually taken as 10% or 20%.

**Figure 3:**
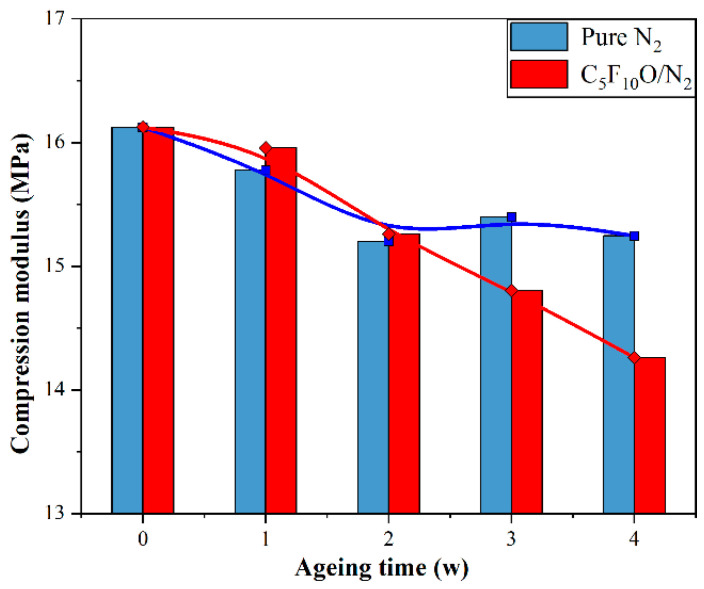
Compressive modulus of aged NBR when *ɛ* = 10%.

Figure 3 illustrates the changes in compressive modulus of NBR after thermal acceleration aged in pure N_2_ and C_5_F_10_O/N_2_ mixture at 100 °C. From Figure 3, the compressive modulus of NBR aged in N_2_ declined in the first week, but in the second to fourth weeks of the test the compressive modulus of NBR was basically stable. This is because N_2_ does not react with NBR, so the compressive modulus of NBR aged in N_2_ will not continue to deteriorate after the movement of the molecular chain at the initial stage.

On the contrary, the compressive modulus of NBR in the C_5_F_10_O/N_2_ showed a significant decrease, and the decline rate showed an acceleration trend with the increase in ageing time. This result demonstrates that NBR has poor compatibility with C_5_F_10_O and may interact with C_5_F_10_O during their long-term coexistence. The interaction will lead to continuous deterioration in the mechanical performance of NBR. According to the decrease curve of the compressive modulus, this deterioration is still not saturated within 28 days.

### 3.2. Surface Morphology and Element Content of NBR

To find out the interaction mechanism between NBR and C_5_F_10_O, scanning electron microscope (SEM) and energy dispersive spectrometer (EDS) were used to investigate the morphology and element content on the surface of NBR before and after the thermal ageing. The tests are performed using a Zeiss GeminiSEM 500 microscope made by Carl Zeiss, Tübingen, Germany, which is equipped with the EDS module of Oxford UltimMax 65 made by Oxford Instruments PLC, Oxford, England.

**Figure 4:**
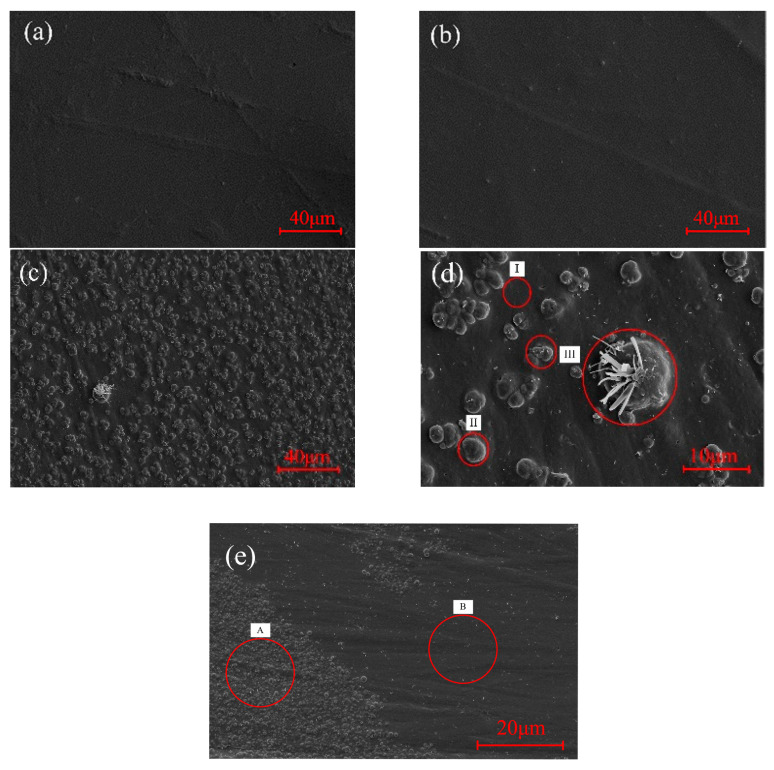
Surface morphology change of (**a**) original NBR, (**b**) NBR aged in N_2_, (**c**) NBR aged in C_5_F_10_O/N_2_ magnified 500 times, (**d**) NBR aged in C_5_F_10_O/N_2_ magnified 2000 times and (**e**) NBR aged in C_5_F_10_O/N_2_ magnified 100 times.

Figure 4 shows the surface morphology of the NBR samples after different ageing conditions. From Figure 4a,b, the surface of NBR aged in N_2_ remains as smooth as the original NBR. While Figure 4c shows that there is an obvious change on the surface of NBR after two weeks’ thermal ageing in C_5_F_10_O/N_2_. In Figure 4c, a large number of protrusions are densely distributed on the rubber surface. By magnifying the surface of NBR 2000 times, Figure 4d clearly presented the three forms of the NBR’s surface: I. smooth surface without foreign matter; II. slightly raised surface wrapping crystals inside; III. rubber with large protrusions and dendritic or dot-like crystals on the surface.

**Figure 5:**
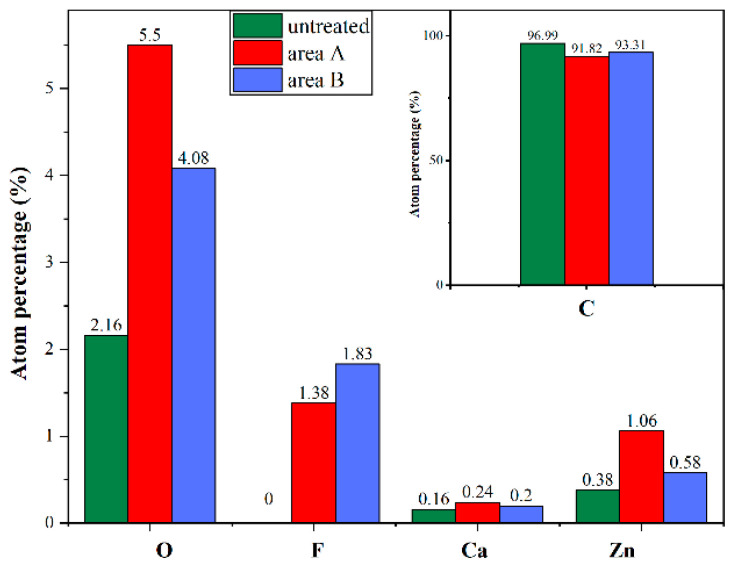
Comparison of element contents on the untreated NBR surface, area A and area B.

The different forms of the surface morphology may be related to the degree of interaction between NBR and C_5_F_10_O. To further clarify the deterioration behaviors of the NBR, EDS was applied to distinguish the element content on the raised surface (area A) and flat surfaces (area B) of the experimental group illustrated in Figure 4e. The element content distribution of aged surfaces compared to the untreated NBR surface is given in Figure 5.

It is apparent in Figure 5 that F atoms that are not initially present in untreated NBR are detected in both area A and B, which directly confirms that NBR reacted with C_5_F_10_O and F atoms in C_5_F_10_O are transferred to the rubber surface during the reaction. Compared to the untreated NBR, the aged surface contains more O, Ca, Zn atoms and less C atoms. Meanwhile, the amount of O, Ca and Zn in area A is significantly larger than that in area B, and the growth of these elements caused a slight decrease in the proportion of F and C atoms in area A. This indicates that protrusions appearing on the aged NBR’s surface contain more ZnO and CaCO_3_, and proves that the crystals observed in Figure 4d could be the reinforcing agent inside NBR which migrated to the surface during the thermal ageing.

Combined with the above analysis, the deterioration process of NBR caused by interaction with C_5_F_10_O can be divided into three stages according to forms of surface morphology:

I. Preliminary reaction. In the long-term contact with C_5_F_10_O, copolymer of butadiene and acrylonitrile starts to react with C_5_F_10_O, resulting in a decrease in the elasticity and strength on the partial surface. However, the effect is not significant in the initial stage, so the rubber surface remains smooth.
II. Additive migration. As the surface of NBR continues deteriorating, it is easier for the reinforcing agent inside the rubber to come out of the less-strength surface during its immigration urged by heat. This is also why bulges wrapping ZnO or CaCO_3_ crystals were found on the NBR surface.
III. Additive precipitation. As the elasticity and hardness of the rubber surface continue to decrease, more and more additives migrate to the surface of the rubber and then pierce the weakened surface, forming observable dot-like crystals. After that, the additives begin to extend on the damaged surface and finally form dendritic crystals on the protrusions, whose volume is also greatly enlarged. This process further promotes the deterioration of the rubber’s mechanical performance.

## 4. Deterioration Mechanism Analysis of NBR Aged in C_5_F_10_O/N_2_ Mixture

To ascertain the reaction on the rubber surface and the mechanical properties change brought by the reaction, the possible interaction between NBR and C_5_F_10_O molecule is analyzed based on density functional theory. Then, the simulated microstructures of aged and untreated NBR are constructed in molecular dynamics simulations. Finally, the impact of the interaction on the mechanical properties of rubber is analyzed.

### 4.1. Reaction Mechanism of NBR and C_5_F_10_O

#### 4.1.1. Primary Decomposition of C_5_F_10_O

Existing research has basically revealed the pathways and thermodynamic properties of C_5_F_10_O decomposition reactions. The decomposition of C_5_F_10_O will generate natural molecules and free radicals such as C_3_F_8_, CF_4_, C_2_F_6_, CF_3_CFCOCF_3_, COF, CF_3_ and CF_2_. However, in the compatibility experiment, the rate and equilibrium constants of these reactions are limited by the applied temperature of 100 °C. Therefore, primary decomposition reactions of C_5_F_10_O with lower energy barrier are more likely to occur. The following three reactions, R1–R3, have the lowest energy barriers of 61.43 kcal/mol, 64.26 kcal/mol and 67.40 kcal/mol in the primary decomposition pathways of C_5_F_10_O, and hence are the dominant reactions that may occur in the experiment [9,28,29].

(2) $$R1:C_{5}F_{10}O\to CF_{3}CCFCF_{3}•+CF_{3}CO•$$

(3) $$R2:C_{5}F_{10}O\to CF_{3}CFCOCF•+CF_{3}•$$

(4) $$R3:C_{5}F_{10}O\to F_{3}CCF(CO)CF_{3}•+CF_{3}•$$

**Figure 6:**
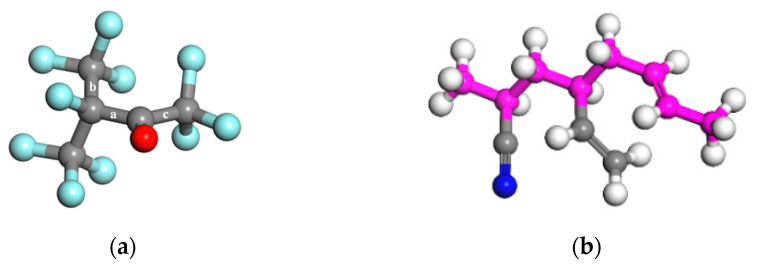
Molecule structure of (**a**) C_5_F_10_O and (**b**) typical repeat unit of NBR rubber. (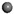
C, 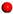
O, 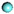
F; 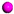
C in backbone, 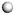
H, 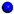
N. The sequence in the repeat unit is acrylonitrile, 1,2-addition butadiene, 1,4-addition butadiene.)

The three primary decomposition reactions correspond to the breaking of C-C bonds marked a, b and c in the C_5_F_10_O molecular shown in Figure 6a. Due to higher energy being required to continue decomposing, F_3_CCFCF_3_, CF_3_CFCOCF_3_ and F_3_CCF(CO)CF_3_ produced by the primary reactions can hardly decompose into smaller radicals. Nevertheless, the decomposition of C_5_F_10_O by R1, R2 and R3 will all generate CF_3_. R1 can generate CF_3_ because CF_3_CO will subsequently decompose into CF_3_ and CO by R4 with an energy barrier of only 11.2 kcal/moL [30]. Therefore, the main small radical generated in the compatibility experiment is CF_3_, which provides the possibility for reactions on the NBR surface.

(5) $$R4:CF_{3}CO•\to CO+CF_{3}•$$

#### 4.1.2. C=C Double Bonds in NBR

Copolymer consisting of NBR is formed via the polymerization of butadiene (CH_2_=CH–CH=CH_2_) and acrylonitrile (CH_3_=CH–CN). Butadiene can be attached to the backbone chain by 1,2 or 1,4 addition reactions during the polymerization. Figure 6b illustrates a typical repeat unit of NBR. When polymerizing into a copolymer chain, each butadiene molecule will leave a reactive C=C bond on the main chain or branched chain of NBR, where addition reactions of small radicals are prone to occur. Consequently, the product CF_3_• generated by the decomposition of C_5_F_10_O can be bonded to the unsaturated C atoms in the copolymer, causing the deterioration of the rubber.

To verify the possibility of this reaction, we calculated the energy barrier and enthalpy of the addition reactions based on the density functional theory in the Dmol^3^ module in the Material Studio [31]. The B3LYP functional applicable to the C/H/O/F molecular system is applied to optimize the initial structure of the reactants and products [11]. The convergence thresholds of the maximum force and the maximum displacement in the structural optimization are set to 0.004 Ha/A and 0.005A, respectively. Then the enthalpy of the reactions is calculated on the same theoretical level.

**Figure 7:**
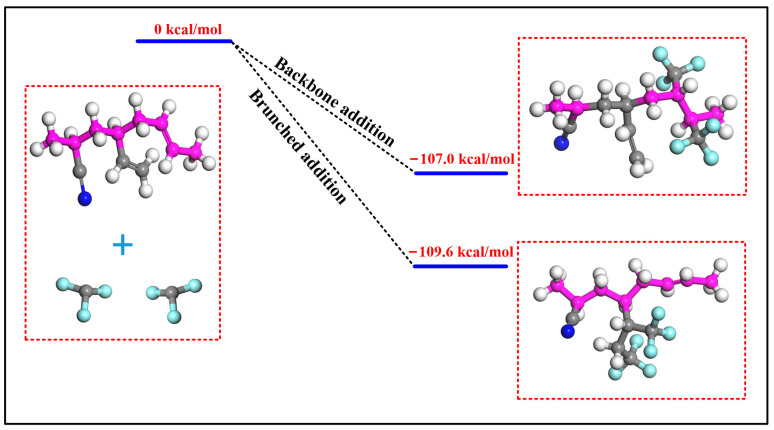
Enthalpy of the addition reactions at B3LYP level.

As shown in Figure 7, addition reactions of CF_3_ bonded to the main and branch chain of the copolymer molecules are both exothermic reactions without transition states, and the enthalpies are −107.0 kcal/moL and −109.6 kcal/moL, respectively. The results indicate that the addition reactions easily occur even at room temperature. Therefore, once CF_3_ is produced through the decomposition of C_5_F_10_O, it can readily react with the unsaturated bonds in NBR via addition reactions. Meanwhile, although the primary decomposition reactions can hardly cause considerable decomposition of C_5_F_10_O under the limitation of the equilibrium constant at 100 °C, when coexisting with NBR, CF_3_ in the decomposition products will react with NBR and gradually be consumed, making the reaction equilibrium continue to move forward. Compared to the experimental results in Figure 5, the addition reactions also explain why F atoms that do not exist in untreated NBR can be found on the surface of aged NBR in the EDS tests.

### 4.2. Simulation of Rubber Mechanical Properties Based on Molecular Dynamics

#### 4.2.1. Model Building

Based on the analysis in Section 4.1, an NBR cell with periodic boundary conditions is established in this section. The influence of the interactions between C_5_F_10_O and NBR on the mechanical properties of NBR is analyzed through the adjustment of molecular structure before and after the thermal ageing. The establishment, structural optimization and mechanical performance calculations of the NBR cells are all carried out under the Condensed-phase Optimized Molecular Potentials for Atomistic Simulation Studies (COMPASS) force field [32,33] in Materials Studio. Before calculating the mechanical properties, the following steps are conducted to ensure that the structure of each cell is fully relaxed and optimized so as to make it closer to the actual state of the NBR [34,35].

Firstly, the structure of the amorphous cell is optimized to its minimum energy. Then 20 cycles of dynamic annealing from 453K to 298K is performed under the NVT ensemble, simulating the process of cooling the NBR from the manufacturing temperature to room temperature. The annealed structure finally undergoes two dynamic relaxations of 500 ps under the NPT and NVT ensemble, respectively. In the calculation, the Ewald method is selected for electrostatic interaction, the Atom Based method is selected for Van der Waals force, the Nose–Hoover thermal bath method is used for temperature control and the Berendsen method is used for pressure control.

**Figure 8:**
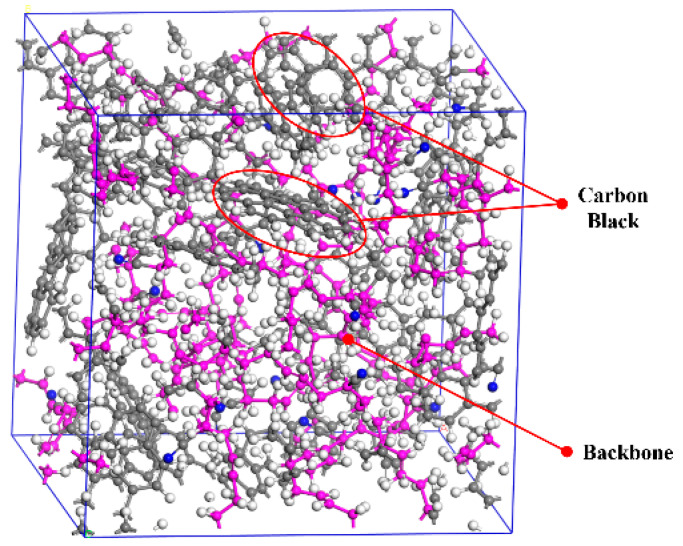
Unit cell model of NBR rubber.

Carbon black and copolymer molecular chains are selected as the two main components of the NBR simulation model based on the proportions given in Table 1. According to [36], when the degree of polymerization of the molecular chain exceeds 10, the calculated results of its mechanical and chemical properties will be generally consistent with the actual situation. Thus, there are five copolymer molecular chains in the amorphous cell, where the degree of polymerization of each molecular chain is set to 20 and the ratio of butadiene to acrylonitrile is 4:1. The carbon black with a microcrystalline structure is composed of two layers of C atoms arranged in a regular hexagon manner, with the layer spacing and the C-C bond length of 334.8 pm and 142 pm. The number of carbon black molecules in the cell is determined by the mass ratio polymer to carbon black (5:4) measured in the thermogravimetric test. Figure 8 illustrates the original NBR cell in the simulation.

#### 4.2.2. Deterioration Mechanism of NBR

By applying small stresses in six directions on the optimized cell, the strain under different stress can be obtained, and the elastic coefficient matrix of the amorphous can be calculated accordingly [37]. Based on the isotropy assumption, the elastic coefficient matrix of NBR is as follows:

(6) $$C=\left[\begin{array}{cccccc} 2\mu +\lambda & \lambda & \lambda & 0 & 0 & 0\\ \lambda & 2\mu +\lambda & \lambda & 0 & 0 & 0\\ \lambda & \lambda & 2\mu +\lambda & 0 & 0 & 0\\ 0 & 0 & 0 & \mu & 0 & 0\\ 0 & 0 & 0 & 0 & \mu & 0\\ 0 & 0 & 0 & 0 & 0 & \mu \end{array}\right]$$

where *λ* and *μ* are called Lame constants and determine the strain–stress relationship of the material. The material’s elastic modulus E, shear modulus G and bulk modulus B can all be calculated via *λ* and *μ*:

(7) $$E=\mu \frac{3\lambda +2\mu }{\lambda +\mu }$$

(8) G=μ

(9) $$B=\lambda +\frac{2}{3}\mu$$

Since there is no O_2_ in the closed experimental environment, the possible structural changes of carbon black in rubber are ignored in the simulation. In order to study the influence mechanism of the extent of the reaction on the mechanical properties of NBR, the proportion of C=C double bonds added with CF_3_ on the molecular chain is set to 0%, 25%, 50% and 75%.

**Figure 9:**
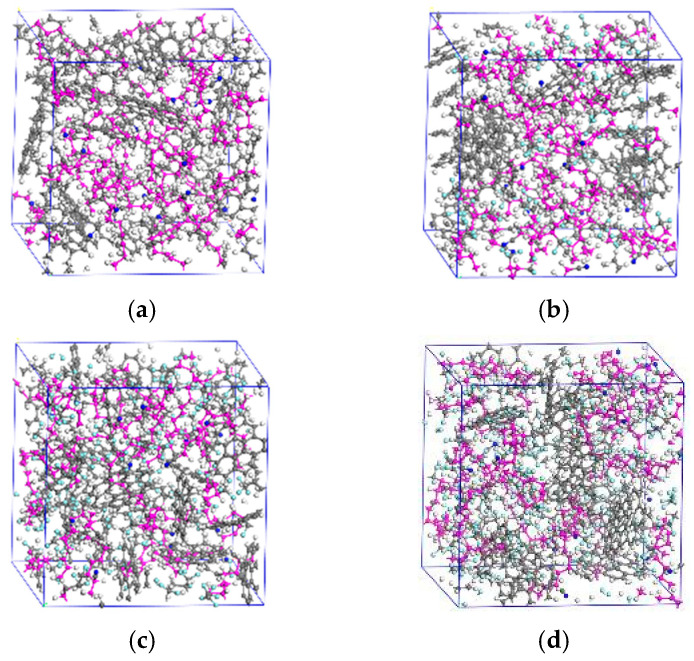
NBR cell with different addition ratios. (**a**) 0%, (**b**) 25%, (**c**) 50%, (**d**) 75%.

**Figure 10:**
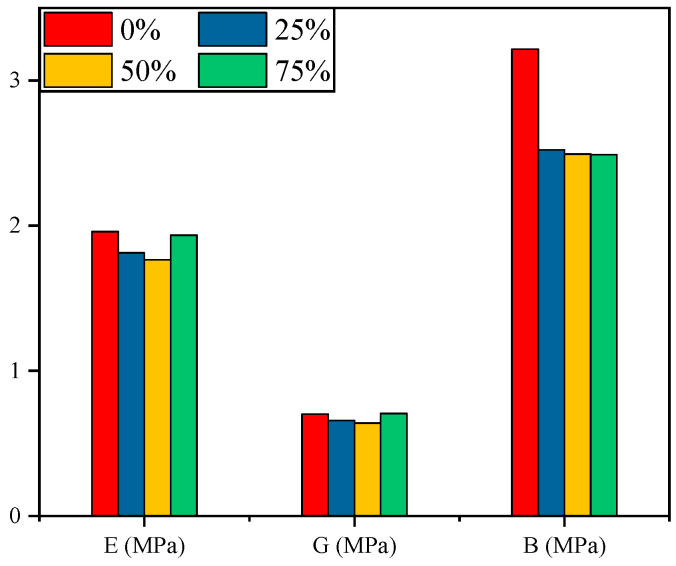
Elastic parameters of NBR cells.

Figure 9 shows the microstructure of the rubber fully relaxed under the four conditions. The elastic parameters of the four structures are calculated through (7)– (9) and are shown in Figure 10. The elastic modulus calculated in the simulation corresponds to the compression modulus presented in Figure 3. Compared to the results of compressive modulus tests, the calculated elastic modulus is smaller than the compression modulus measured in the actual test. This is because ZnO, CaCO_3_ and other reinforcing agents will be added to the rubber during the actual manufacturing process, which greatly increases the macroscopic strength of NBR.

From Figure 10, when the amount of added C=C double bond reaches 25%, a small number of new branches is attached to the carbon chain of the NBR surface, which increases the free volume in the cell and makes it more susceptible to deformation when subjected to external forces. Hence the elastic modulus, shear modulus and bulk modulus of the aged NBR all decreased significantly compared to the original NBR. This trend is consistent with the changes in the compression modulus obtained experimentally. However, as the proportion of added C=C double bonds continues to increase, these newly grafted CF_3_• on the carbon chain fill the free volume caused by the aforementioned addition reaction, and the rubber structure becomes denser. Therefore, when the amount of added C=C double bonds increases to 50%, the decline in the elasticity parameters is reduced.

When the amount reached 75%, the elastic modulus and shear modulus showed an increasing trend. In the compression modulus test, NBR rubber did not show a reverse trend of mechanical properties during the continuous ageing process. This is because when the surface strength of the NBR weakened at the initial stage of deterioration, the internal reinforcing agent had already migrated to the rubber surface and further caused irreversible deterioration of the rubber’s mechanical properties. Consequently, even if the downward trend of the polymer elastic modulus of the rubber surface can be slowed down at the end of the addition reaction process, the rubber properties will not recover again.

While this simulation study provides insights into the effects of C_5_F_10_O on NBR’s elastic properties, there are limitations that should be discussed. As listed in Table 1, real rubber is a complex mixture of organic polymers, additives and other substances. In our simulation, different additives used to regulate the rubber’s production process were not included. Although the proportion of additives is much lower, they may still somehow affect the performance and compatibility of rubber. Therefore, the proposed reaction mechanism in this study might not fully capture the interaction between NBR and C_5_F_10_O, as the possibility of inorganic additives reacting with C_5_F_10_O was not taken into account. Additionally, due to computational limitations, the size of the constructed polymer model was not large enough to match the particle size of real additives, which might affect our understanding of the impact of additives on the system.

## 5. Conclusions

In this paper, the deterioration behaviors of the sealing material NBR coexisting with C_5_F_10_O/N_2_ mixture is studied through thermal accelerated ageing experiment. The deterioration stages of NBR are analyzed based on the obtained compressive modulus, surface morphology and element content. Potential interactions between C_5_F_10_O and NBR are considered, and the resulting effects on the mechanical properties of NBR are then investigated via molecular dynamics simulations. The specific conclusions are as follows:

(1) The compressive modulus of NBR aged in C_5_F_10_O/N_2_ mixture is significantly smaller than that of NBR aged in N_2_, indicating that C_5_F_10_O is incompatible with NBR rubber. Therefore, when in long-term contact with C_5_F_10_O in the electrical equipment, the sealing performance and the service life of NBR will be weakened.
(2) NBR aged in C_5_F_10_O undergoes a three-stage deterioration process based on changes in its surface morphology and atomic composition: (I) a preliminary reaction between NBR and C_5_F_10_O results in the reduction of surface strength; (II) reinforcing agents such as ZnO and CaCO_3_ inside the NBR migrate to the surface, forming bumps that encase crystals on the rubber surface; (III) with further weakening of the surface strength, the reinforcing agents penetrate the rubber surface and exhibit branch-like extensions at the bumps. Each stage is accompanied by a decrease in the mechanical strength of NBR.
(3) DFT and MD simulations suggest that the C=C double bonds in the molecular chain of NBR can react with CF_3_ radicals generated by the primary decomposition of C_5_F_10_O. Then the addition of C=C double bonds and introduction of CF_3_ groups on the molecular chain will cause a decrease in the elastic, shear and bulk modulus of NBR. This results in the internal reinforcing agents precipitating onto the surface of NBR, thereby further intensifying the irreversible deterioration of its mechanical properties.

## Figures and Tables

**Table 1:** Main components of NBR used in the experiment.

Composition	Weight Fraction (%)	Temperature (°C)
Oil	5.8	200~380
Polymer	49.0	390~500
Carbon black	39.8	527~600
CaCO_3_	0.38	610~637
ZnO/MgO	4.14	>800

## Data Availability

Not applicable.
